# Evaluation of a dementia awareness game for undergraduate nursing students in Northern Ireland: a Pre-/Post-Test study

**DOI:** 10.1186/s12912-023-01345-2

**Published:** 2023-05-22

**Authors:** Stephanie Craig, Patrick Stark, Christine Brown Wilson, Gillian Carter, Sonya Clarke, Gary Mitchell

**Affiliations:** School of Nursing and Midwifery, Queen’s University Belfast, Belfast, Northern Ireland

**Keywords:** Dementia, Nurse education, Nursing student, Serious game, Pedagogical research, Constructivist pedagogy, Quantitative research methods

## Abstract

**Introduction:**

Although it is possible to live well with dementia and many individuals with dementia lead active lives with the help of family, friends, and communities, the general impression of dementia is frequently negative. Dementia is a global health issue. Despite this, little research has been done on the effects of innovative dementia education strategies among undergraduate nursing students. The aim of this study was therefore to assess if this serious digital game, originally intended for the public, could increase knowledge about dementia in first-year nursing students.

**Methods:**

The intervention was a digital serious game called “The Dementia Game”, which was available to students throughout February 2021, to a convenience sample of first-year undergraduate nursing students (n = 560) completing a BSc Honours Nursing Degree programme in one university in Northern Ireland. The game was evaluated using a pretest-posttest design. The questionnaire comprised of a 30- item true- false Alzheimer’s Disease Knowledge Scale (ADKS), which covers risk factors, assessment and diagnosis, symptoms, course, life impact, caregiving and treatment and management. Data were analysed using paired t-tests and descriptive statistics.

**Results:**

Overall dementia knowledge increased significantly after playing the game. Pre-test to post-test increases were observed across a range of seven categories of dementia knowledge (life impact, risk factors, symptoms, treatment, assessment, caregiving and trajectory), with particularly large increases in knowledge of trajectory and risk factors, as shown using paired t-tests. All pre-test to post-test comparisons were significant at the p < 0.001 level.

**Conclusions:**

A short serious digital game on dementia improved first-year student’s knowledge about dementia. Undergraduate students also expressed that this approach to dementia education was effective in improving their knowledge about the disease.

**Supplementary Information:**

The online version contains supplementary material available at 10.1186/s12912-023-01345-2.

## Introduction

There are over 10 million new cases of dementia each year, with more than 55 million people living with dementia worldwide [1]. Dementia is an umbrella term [2] that encompasses a wide spectrum of progressive neurological illnesses, with over 200 subcategories. Clinical manifestations of dementia, although unique to everyone, generally present as deterioration of memory, thinking and behaviour which accordingly limits the individual to perform daily activities. Alzheimer’s Disease has been identified as the most common form of dementia, contributing to 60–70% of cases diagnosed, while other types of dementia include vascular dementia, Lewy Body dementia and frontotemporal dementia [1]. Due to the increased prevalence of dementia, it is important that health professionals know about the condition to improve their caring abilities to enhance the care of the person living with dementia.

Nurses play a significant role in primary, secondary, and tertiary care and are likely to support individuals living with dementia from pre-diagnosis to end-of-life care because they are the largest single group of healthcare professionals worldwide [3–6]. Subsequently, throughout the course of their diagnosis, people who have dementia will also be supported by nursing students who are the future workforce of healthcare professionals [7]–[8]. Student nurses need to be equipped through dementia education to be able to help, support and care for people living with dementia whom they will meet through clinical placements in their nursing training [9]. Nurse education is always evolving; a new innovative way of healthcare education is gamification or the use of ‘serious games’.

Gamification is a concept that incorporates gaming elements in education to enhance levels of student engagement within an educational environment. The use of ‘serious games’ is an increasingly effective method in educating health professionals, and improving engagement, user retention, knowledge, and cooperation [10]–[11]. Serious games are a computer-delivered intervention used to support players to learn information and practice their skills through gaming [12]. A recent systematic review and meta-analysis concluded that digital gaming can be as effective as a stand-alone or a multi-component program while appealing to a diverse population regardless of age or gender [10]. A prominent instructional method in the field of nursing education is the use of serious games [12–17]. Supporters of digital games also promote the accessibility and convenience of such games [18]. A fresh approach to learning and increasing comprehension is provided by serious games, for example, dementia knowledge could be improved via serious games in nursing education. To the author’s knowledge, there has been no empirical research carried out on the use of a digital serious game to improve undergraduate student nursing knowledge about dementia. Therefore, research in this area could help to establish whether serious games are a useful tool in this context. A recent review of the literature by Malicki [17] and colleagues on the use of gamification in nursing education found that interactive digital learning, including games, gamification, and scenario-based learning, can positively impact learner engagement and satisfaction. However, the studies reviewed did not provide measurable evidence regarding knowledge acquisition.

The study authors previously co-designed a serious digital game to improve public perception of dementia (www.dementiagame.com) [19]. While there are many ways to educate student nurses about dementia, this study therefore aimed to assess if this serious digital game, originally intended for the public, could increase knowledge about dementia in first-year nursing students by using a pre-/post-test design.

## Methods

### Design/ setting/ population

A quantitative pre-test/post-test design was used to compare nursing students’ knowledge about dementia before and after playing the Dementia Awareness game. During the period of February 2021, a convenience sample of first-year adult undergraduate nursing students from Queen’s University Belfast in Northern Ireland was sampled to take part in the study. At the time of this study, all eligible nursing students had completed clinical placement time in a care setting, but they had not received structured university education (for example a lecture, tutorial, or reading) on dementia, which traditionally comes in years two and three of their programme. Table 1. Highlights inclusion/ exclusion criteria used in this study.

**Table 1:** Inclusion/ exclusion criteria for participants

Inclusion criteria:	Exclusion criteria:
♣ Students who are current enrolled in their first year at Queen’s University Belfast, are studying adult nursing and are part of the February 2020 cohort.	♣ Students have not played the dementia awareness game.
♣ Students who have played the dementia awareness game as part of their module learning.	♣ Students not enrolled in their first year at Queen’s University Belfast, are not studying nursing and are not part of the February 2020 cohort.

### Intervention

A digital serious game called “The Dementia Game” was previously developed by the study authors [19] following codesign with people living with dementia alongside stakeholder engagement [20]. The serious game works on any device with an internet connection and is a web-based application (HTML5). Players must follow a path to the finish line and do so by responding to a series of multiple-choice questions about dementia that are presented in a random sequence from an existing question bank. These questions were jointly created with people living with dementia and designed to dispel common misconceptions and test the players’ knowledge, attitudes, and behaviours towards dementia. Correct responses to questions result in points while crossing the finish line earns additional points. Players can challenge others to a game and record their scores. The “Dementia Game” lasts for around 90 s, and participants can play as many times as they choose.

### Survey instrument

The 30-item Alzheimer’s Disease Knowledge Scale (ADKS) was utilised [21]. This online questionnaire which was emailed to participants can be viewed in Supplementary File 1. The true/false scale takes approximately 5–10 min to complete and covers risk factors, assessment and diagnosis, symptoms, course, life impact, caregiving and treatment and management. This validated tool has been used in the evaluation of dementia knowledge in healthcare professionals, the general public and student nurses [22–24]. All students including those who did not take part in the study received a copy of the ADKS and the right answers once data collection was finished to reinforce what had been learned. No items of the survey instrument were presented in the serious game.

### Consent/ recruitment

An individual unaffiliated with the project sent an email to all eligible participants (n = 560) informing them of the study and the opportunity to take part. It was made clear that their choice to participate in this study was voluntary and would have no bearing on their course progression or module grades. Students who participated in the surveys and used the game required access to their own laptop, tablet, or phone. Given the nature of the intervention, a digital serious game, it was not possible for participants to engage with a non-digital version of the game nor complete questionnaires on paper.

### Data collection (Fig. 1.)

The pre-and post-questionnaires were completed by the students who chose to take part in the study within the same four-week period that they had access to the game. Participants received three weblinks at three-time points. The first time point was for the pre-test and participants could complete the pre-test questionnaire at any time over a seven-day period. The second time point occurred after the pre-test closed. All participants that completed the pre-test and provided online informed consent to obtain the game, were emailed a link to the serious game. Participants were given four weeks to play the game as often as they wished. A reminder email was sent to all participants about how to access the game at a midway point (at the end of week two). Following this four-week period, access to the game was closed and participants were sent their post-questionnaire to complete. The post-test was open for a period of fourteen days, with a reminder email sent midway (at the end of week one).

**Fig. 1:**
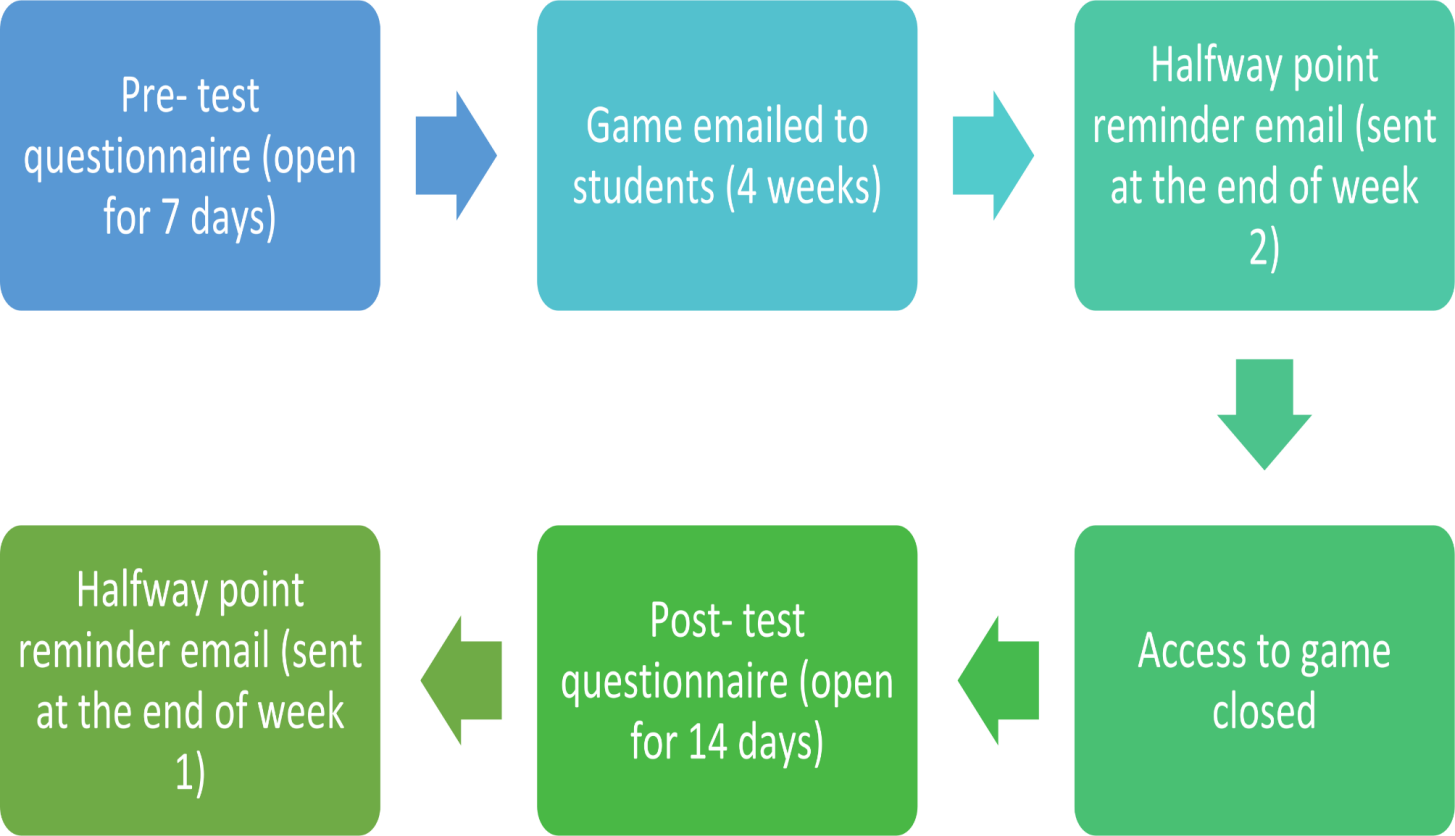
Data collection flow chart

### Ethics

This study received ethical approval by Queen’s University Belfast’s Faculty of Medicine, Health, and Life Sciences Research Ethics Committee (MHLS 20_61). Online informed consent was obtained from all questionnaire participants. The study took place between February 2021 and August 2021. All methods were performed in accordance with the Declaration of Helsinki [25].

### Data analysis

The pre and post test datasets were matched prior to analysis. Matching was carried out using student ID numbers which served as an identifier for each participant between the two datasets. All analyses were conducted in SPSS version 27.

Scores were calculated as the percentage of correct answers for pre-test and post-test total scores and sub-scales. Total percentage scores for each participant were calculated by summing response scores for each item, dividing by the number of items, and multiplying by 100. Sub-scale percentage scores were calculated using the same method, but only scores for items associated with that sub-scale were summed, then divided by the number of items in that sub-scale (see Table 2 for number of items in each sub-scale) All analyses were conducted using the percentage-based scores. Descriptive statistics were calculated for pre-test and post-test scores. Distribution of pre-test scores was examined using a histogram to investigate any floor or ceiling effects or potential for regression to the mean when comparing with post-test scores. A paired t-test was conducted to examine the change from pre-test to post-test for total scores. Seven paired comparisons were conducted using paired t-tests for pre-test and post-test scores for the seven sub-scales (life impact, risk factors, symptoms, treatment, assessment, care giving and trajectory).

In total, eight comparison analyses were conducted during this study. A Bonferroni correction was applied to the alpha value when determining the statistical significance of the results of these analyses to reduce the risk of false positives associated with multiple comparisons [26]. Alpha (0.05) was divided by the total number of comparisons in this study (8) to give an alpha value of *α* = 0.006. Results of the pairwise comparisons in this study, therefore, were only considered to be statistically significant if their associated p-value was 0.006 or below.

## Results

Raw scores for the seven sub-scales (life impact, risk factors, symptoms, treatment, assessment, care giving and trajectory) were also converted to percentages in SPSS. A set of seven paired comparisons using pre-test and post-test scores for these subscales was conducted as seen in Table 2.

**Table 2:** Seven Subscales and corresponding questions

Number:	Subscale:	Related question from ADKS:
1.	Life Impact	28,11,1
2.	Risk Factor	27,25,13,2,18,26
3.	Symptoms	19,30,23,22
4.	Treatment	29,24,9,12
5.	Assessment	10,21,20,4
6.	Care Giving	5,15,7,16,6
7.	Trajectory	8,17,3,14

### Missing data

560 students were eligible to participate in the study. A total number of 452 participants out of 560 possible participants responded to at least one of the survey time-points in the study, i.e., pre-test, post-test or both time-points. N = 410 participants completed pre-testing. Non-participation at pre-test stage was either due to not participating in the study (n = 108) or participating but failing to complete a pre-test (n = 42). N = 376 completed post-testing, meaning that n = 76 participated in the study but did not complete a post-test. Primary analysis was possible for n = 334 participants out of the total of 452 participants due to missing data. If a participant was missing either pre-test or post-test data, they were not able to be included in the dependent t-test analysis for the total ADKS score, but their non-missing data was included in the descriptive statistics. Further missing data occurred in the paired comparisons due to participants not supplying a correct identifier (name or student number) to allow their pre-test and post-test data to be matched, despite completing the survey. These missing data can be seen in Table 3.

**Table 3:** Missing data

	Missing n (%)
Pre-test data	42 (9.3%)
Post-test data	76 (16.8%)
Paired-comparisons	118 (26.1%)

### Primary analysis results

**Fig. 2:**
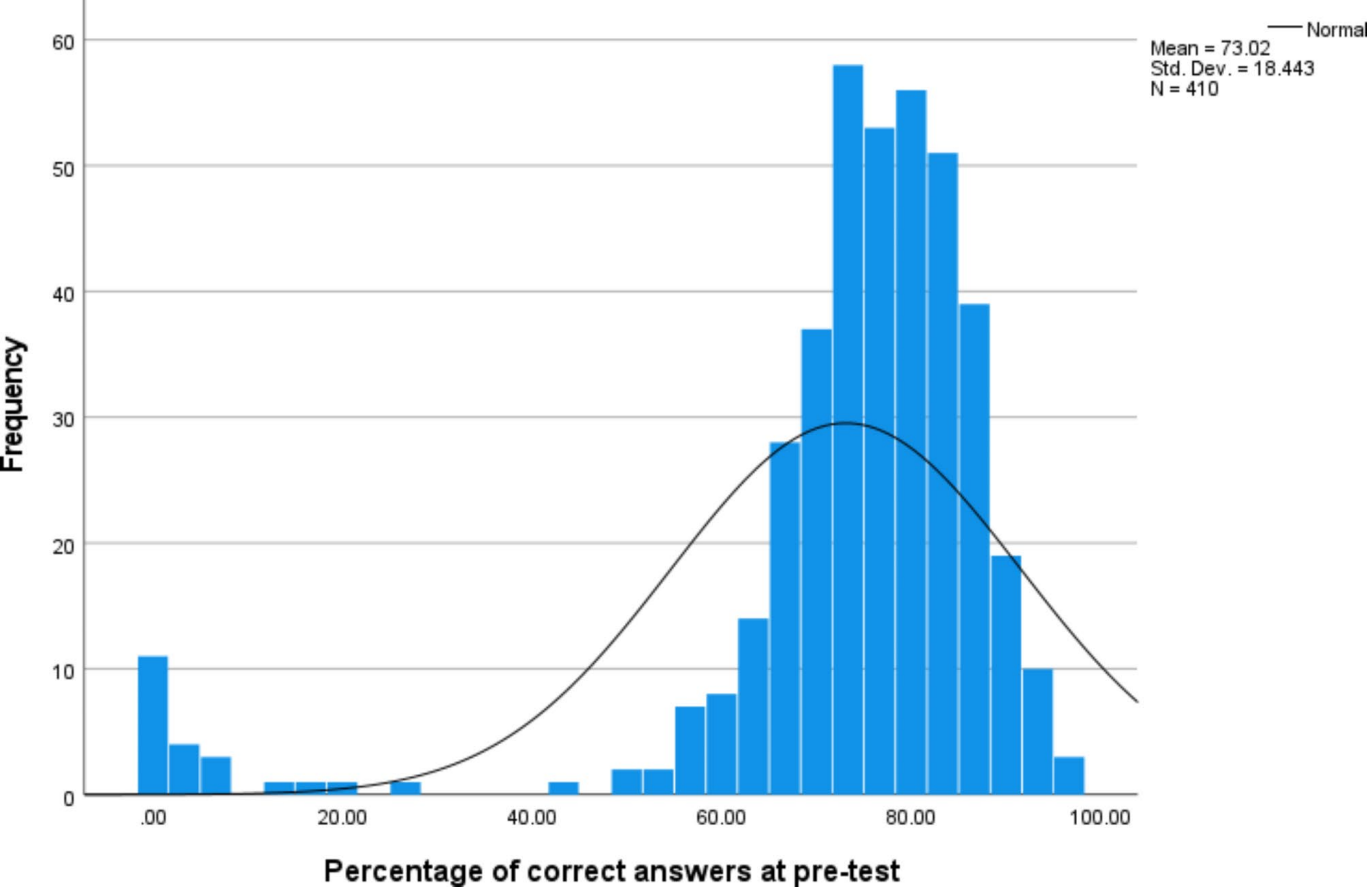
Distribution of pre-test scores

The distribution of pre-test scores (Fig. 2) shows that there is an approximately normal distribution, with no strong positive or negative skew or evidence of ceiling or floor effects.

Descriptive statistics (Table 4) shows that post-test scores (M = 89.40, SD = 15.08) were higher than pre-test scores (M = 73.02 SD = 18.44). The difference between these two scores was statistically significant as indicated by the paired samples t-test which gave a result of t(333) = 20.48, p < 0. 001.

**Table 4:** Descriptive statistics for pre-test and post-test total scores

	N	Range	Minimum	Maximum	Mean	Std. Deviation
TotalScore_pre	410	96.67	0.00	96.67	73.02	18.44
TotalScore_post	376	100.00	0.00	100.00	89.40	15.08
Valid N (listwise)	334					

### Secondary analysis results

Descriptive statistics showed that life impact, risk factors, symptoms, treatment, assessment, care giving, and trajectory knowledge sub-scale scores increased from pre-test to post-test (see Table 5). Minimum and maximum scores collected for all sub-scales were 0 and 100 respectively (removed from table for clarity). In the paired comparison analyses, the largest increases from pre-test to post-test were in the sub-scales for trajectory (23% points) and risk factors (19% points), but all showed a significant increase (see Table 6). All comparisons were statistically significant at a level of p < 0.001, i.e., below the Bonferroni-corrected alpha cut-off of p = 0.006.

**Table 5:** pre-test (n = 410) and post-test (n = 376) mean percentage scores for sub-scales

	Pre-test Mean	Pre-test Std. Deviation	Post-test Mean	Post-test Std. Deviation
Life Impact	75.04	26.12	90.96	18.57
Risk Factors	66.95	23.06	87.28	19.01
Symptoms	70.91	25.40	87.43	20.98
Treatment	74.45	23.61	87.70	19.32
Assessment	79.39	25.19	89.03	19.69
Care Giving	79.32	23.68	93.09	17.44
Trajectory	67.07	26.42	90.82	19.03

**Table 6:** Paired comparison t-tests for pre-test and post-test sub-scale percentage scores (n = 334)

		Mean increase from pre-test to post-test	Std. Deviation	t^a^	df^b^	Sig. (2-tailed)
Pair 1	Post-test life impact and pre-test life impact	14.77	25.73	10.49	333	< 0.001
Pair 2	Post-test risk factors and pre-test risk factors	18.81	22.06	15.58	333	< 0.001
Pair 3	Post-test symptoms and pre-test symptoms	15.34	23.46	11.96	333	< 0.001
Pair 4	Post-test treatment and pre-test treatment	11.45	21.79	9.61	333	< 0.001
Pair 5	Post-test assessment and pre-test assessment	7.26	20.27	6.55	333	< 0.001
Pair 6	Post-test care giving and pre-test care giving	12.28	19.76	11.35	333	< 0.001
Pair 7	Post-test trajectory and pre-test trajectory	23.13	25.60	16.51	333	< 0.001

^*a*^ *paired samples t-test*

^*b*^ *degrees of freedom*

## Discussion

This study demonstrated a significant increase in overall dementia knowledge following the game-based intervention. The results showed a substantial improvement in knowledge across various domains, including life impact, risk factors, symptoms, treatment, assessment, caregiving, and trajectory of dementia. Notably, the participants exhibited particularly large gains in knowledge of the trajectory and risk factors of dementia, which was confirmed by paired t-tests. These findings suggest that game-based interventions have the potential to improve dementia knowledge among nursing students, as demonstrated by the significant increase in knowledge observed in the study. The results also suggest that incorporating game-based interventions in dementia education may be an effective strategy to improve knowledge retention in this population. Further research in this area could provide valuable insights into the effectiveness of game-based interventions in dementia education, as well as their potential to enhance the quality of care provided by nursing students to individuals with dementia.

Previous research has suggested that asynchronous learning can pose challenges for students due to conflicting schedules and external commitments, leading to difficulty in keeping up with the volume of content presented to them [27]. Moreover, students may feel pressure to keep pace with asynchronous content, potentially impacting their self-regulated learning behaviours and motivation [28]. In the context of COVID-19, many studies have highlighted the difficulty students face in engaging in self-regulated learning experiences, which can result in distraction and disengagement [27, 29−31]. However, this study, an asynchronous serious game about dementia for nursing students, demonstrated a positive impact of asynchronous learning evidenced by knowledge acquisition. This may be attributed to the engaging and informative nature of the serious game, which kept students motivated and interested in learning. It may also be because the serious game was originally co-designed by people with dementia and nursing students and therefore could be more meaningful as it included the ‘lived experience’ of dementia [19].

Serious games or gamification have emerged as a popular instructional strategy in nurse education, and their use has been noted in several professional fields [32–35]. From a pedagogical perspective, gamification, or the use of serious digital games, provides a viable alternative to traditional teaching techniques as a means of enhancing knowledge acquisition [36]. The convenience and accessibility of serious digital games are also cited as advantages [37]. In addition, digital games that are relatively quick to finish have the potential to generate numerous plays and be played by a large audience [32]–[33]. Serious digital games, such as the one offered by www.dementiagame.com, offer a novel approach to learning that can foster motivation, engagement, and the development of learning and problem-solving skills [34]–[35]. Furthermore, gamification has been shown to support behavioural change, for example encouraging nursing students to increase uptake on influenza vaccination [38].

While the use of an asynchronous digital serious game was effective at helping students learn about dementia in the present study, the use of serious games in nurse education does not guarantee successful learning experiences. Indeed, Caserman [39] and colleagues suggest there are few serious games for healthcare education that are underpinned by robust quality standards in consideration to both the intended effect (serious part of the game) and the entertainment effect (game part of the game). While the present study has empirically evaluated the ‘serious part’ of the game, using a validated knowledge questionnaire, the authors have not investigated the extent to which the dementia awareness game is entertaining or could be more entertaining. Further, a recent meta-analysis [40] on the use of serious games in education, recommended that comprehending learners’ attitudes towards serious game-assisted learning is paramount for scholars to devise suitable pedagogical strategies that cater to diverse learner needs, and for practitioners to develop appropriate serious games that enhance learning outcomes. Distinct attitudes reflect the varying needs of learners and practitioners, and it is imperative for serious game developers and instructors to establish a theoretical and practical framework based on these needs. Therefore, future evaluation of serious games should also include other aspects such as entertainment of the game from the student’s perspective, usability of the serious game and the attitude of the student about the use of serious games in education.

### Strengths/ limitations

This study’s notable strength is that, to our knowledge, it is the first of its kind to examine how a co-designed digital dementia awareness game affects first-year nursing student knowledge. A sizable number of students participated, indicating that recruitment for this study was very successful. Additionally, after the intervention, the game significantly increased student nurses’ knowledge of dementia which was captured in the weeks after playing (i.e., not immediately post-test). In this study, all participants were in the same year of nursing school and had completed a clinical rotation of at least six weeks before playing the game. Although this represents a good response rate, the sample was only drawn from one university in Northern Ireland, with no control or comparison group, which restricts the generalizability of the results. Additionally, no demographic information from nursing students was gathered for this study, which could have provided specifics about the participants’ prior medical experience. A limitation of this study is that only knowledge was tested, compared to the previous study [19] on this intervention where only attitude was tested using the 19-item ADQ scale in the public [26]. The rationale for this was that the study authors wanted to determine if knowledge could be acquired through playing a serious digital game. As this has been demonstrated, future research will be required to assess further changes in nursing and other health professions students’ attitudes. Another noted limitation in this study is that the intervention (serious game) cannot guarantee that the new knowledge would translate into better dementia care.

## Conclusion

Serious games have been found to be effective in education. Well-designed serious games aim to foster positive moods to encourage players to continue playing, resulting in increased interest in the gameplay and improved academic performance. Engaging players are more likely to become absorbed in serious game-based learning. The impact of using a serious digital game on students’ knowledge of dementia has been clearly shown in the present study. Overall, there was a statistically significant increase in knowledge about dementia after playing a serious game about the disease. Digital serious games therefore have the potential to reach a large audience including healthcare professions students and appear to be a suitable tool to enhance knowledge of dementia as demonstrated in this study.

## Electronic supplementary material

Below is the link to the electronic supplementary material.

Supplementary Material 1

## Data Availability

The datasets used and/or analysed during the current study are available from the corresponding author on reasonable request.

## Abbreviations

ADKS: Alzheimer’s Disease Knowledge Scale
MHLS: Medicine, Health, and Life Sciences
