# Supplementary material for: Evaluation of a dementia awareness game for undergraduate nursing students in Northern Ireland: a Pre-/Post-Test study
Source: BMC Nurs. 2023 May 22;22:177. doi: 10.1186/s12912-023-01345-2 (PMC10201484; doi:10.1186/s12912-023-01345-2)
Supplement: Supplementary file 1 — Supplementary Material 1 [file 12912_2023_1345_MOESM1_ESM.docx]

**Approaches to Dementia Questionnaire (ADQ)**

***Please note – the participants will not see any numerical ratings associated with their response options (e.g. their options will be Strongly Agree, Agree, Neither Agree nor Disagree, Disagree and Strongly Disagree).***

**1. It is important to have a very strict routine when working with people with dementia.**

| Strongly Agree 1 | Agree 2 | Neither Agree nor Disagree 3 | Disagree 4 | Strongly Disagree 5 |
| --- | --- | --- | --- | --- |

**2. People with dementia are very much like children.**

| Strongly Agree 1 | Agree 2 | Neither Agree nor Disagree 3 | Disagree 4 | Strongly Disagree 5 |
| --- | --- | --- | --- | --- |

**3. There is no hope for people with dementia.**

| Strongly Agree 1 | Agree 2 | Neither Agree nor Disagree 3 | Disagree 4 | Strongly Disagree 5 |
| --- | --- | --- | --- | --- |

**4. People with dementia are unable to make decisions for themselves.**

| Strongly Agree 1 | Agree 2 | Neither Agree nor Disagree 3 | Disagree 4 | Strongly Disagree 5 |
| --- | --- | --- | --- | --- |

**5. It is important for people with dementia to continue to be active and involved in the things they enjoy.**

| Strongly Agree 5 | Agree 4 | Neither Agree nor Disagree 3 | Disagree 2 | Strongly Disagree 1 |
| --- | --- | --- | --- | --- |

**6. People with dementia are sick and need to be looked after.**

| Strongly Agree 1 | Agree 2 | Neither Agree nor Disagree 3 | Disagree 4 | Strongly Disagree 5 |
| --- | --- | --- | --- | --- |

**7. It is important for people with dementia to be given as much choice as possible in their daily lives.**

| Strongly Agree 5 | Agree 4 | Neither Agree nor Disagree 3 | Disagree 2 | Strongly Disagree 1 |
| --- | --- | --- | --- | --- |

**8. Nothing can be done for people with dementia, except for keeping them clean and comfortable.**

| Strongly Agree 1 | Agree 2 | Neither Agree nor Disagree 3 | Disagree 4 | Strongly Disagree 5 |
| --- | --- | --- | --- | --- |

**9. People with dementia are more likely to be contented when treated with understanding and reassurance.**

| Strongly Agree 5 | Agree 4 | Neither Agree nor Disagree 3 | Disagree 2 | Strongly Disagree 1 |
| --- | --- | --- | --- | --- |

**10. Once dementia develops in a person, it is inevitable that they will go downhill.**

| Strongly Agree 1 | Agree 2 | Neither Agree nor Disagree 3 | Disagree 4 | Strongly Disagree 5 |
| --- | --- | --- | --- | --- |

**11. People with dementia need to feel respected, just like anybody else.**

| Strongly Agree 5 | Agree 4 | Neither Agree nor Disagree 3 | Disagree 2 | Strongly Disagree 1 |
| --- | --- | --- | --- | --- |

**12. Achieving a good quality of life for people with dementia involves taking account of their psychological and social needs as well as their physical needs**

| Strongly Agree 5 | Agree 4 | Neither Agree nor Disagree 3 | Disagree 2 | Strongly Disagree 1 |
| --- | --- | --- | --- | --- |

**13. It is important not to get too attached to someone with dementia**

| Strongly Agree 1 | Agree 2 | Neither Agree nor Disagree 3 | Disagree 4 | Strongly Disagree 5 |
| --- | --- | --- | --- | --- |

**14. It doesn’t matter what you say to people with dementia because they forget it anyway.**

| Strongly Agree 1 | Agree 2 | Neither Agree nor Disagree 3 | Disagree 4 | Strongly Disagree 5 |
| --- | --- | --- | --- | --- |

**15. People with dementia often have good reasons for behaving as they do.**

| Strongly Agree 5 | Agree 4 | Neither Agree nor Disagree 3 | Disagree 2 | Strongly Disagree 1 |
| --- | --- | --- | --- | --- |

**16. Spending time with people with dementia can be very enjoyable.**

| Strongly Agree 5 | Agree 4 | Neither Agree nor Disagree 3 | Disagree 2 | Strongly Disagree 1 |
| --- | --- | --- | --- | --- |

**17. It is important to respond to people with dementia with empathy and understanding.**

| Strongly Agree 5 | Agree 4 | Neither Agree nor Disagree 3 | Disagree 2 | Strongly Disagree 1 |
| --- | --- | --- | --- | --- |

**18. There are a lot of things that people with dementia can do**.

| Strongly Agree 5 | Agree 4 | Neither Agree nor Disagree 3 | Disagree 2 | Strongly Disagree 1 |
| --- | --- | --- | --- | --- |

**19. People with dementia are just ordinary people who need particular understanding to fulfil their needs.**

| Strongly Agree 5 | Agree 4 | Neither Agree nor Disagree 3 | Disagree 2 | Strongly Disagree 1 |
| --- | --- | --- | --- | --- |

**TOTAL**

**Hope (Items 1, 2, 3, 4, 6, 8, 10 and 13)**

**Recognition of Personhood (items 5, 7, 9, 11, 12, 14, 15, 16, 17, 18, and 19)**
